# Environmental cleaning and disinfection in the operating room: a systematic scoping review through a human factors and systems engineering lens

**DOI:** 10.1017/ice.2023.280

**Published:** 2024-07

**Authors:** Anping Xie, Hugo Sax, Oluseyi Daodu, Lamia Alam, Marium Sultan, Clare Rock, C. Matthew Stewart, Shawna J. Perry, Ayse P. Gurses

**Affiliations:** 1 Armstrong Institute for Patient Safety and Quality, Johns Hopkins University School of Medicine, Baltimore, Maryland, United States; 2 Department of Anesthesia and Critical Care Medicine, Johns Hopkins University School of Medicine, Baltimore, Maryland; 3 Department of Infectious Diseases, Bern University Hospital and University of Bern, Bern, Switzerland; 4 Johns Hopkins University Bloomberg School of Public Health, Baltimore, Maryland, United States; 5 Division of Infectious Diseases, Department of Medicine, Johns Hopkins University School of Medicine, Baltimore, Maryland, United States; 6 Department of Otolaryngology–Head and Neck Surgery, Johns Hopkins University School of Medicine, Baltimore, Maryland, United States; 7 Department of Emergency Medicine, University of Florida, Jacksonville Medical Center, Jacksonville, Florida, United States; 8 Johns Hopkins Whiting School of Engineering Malone Center for Engineering in Healthcare, Baltimore, Maryland, United States

## Abstract

**Objective:**

To synthesize evidence and identify gaps in the literature on environmental cleaning and disinfection in the operating room based on a human factors and systems engineering approach guided by the Systems Engineering Initiative for Patient Safety (SEIPS) model.

**Design:**

A systematic scoping review.

**Methods:**

Following the Preferred Reporting Items for Systematic Reviews and Meta-Analyses (PRISMA) guidelines, we searched 4 databases (ie, PubMed, EMBASE, OVID, CINAHL) for empirical studies on operating-room cleaning and disinfection. Studies were categorized based on their objectives and designs and were coded using the SEIPS model. The quality of randomized controlled trials and quasi-experimental studies with a nonequivalent groups design was assessed using version 2 of the Cochrane risk-of-bias tool for randomized trials.

**Results:**

In total, 40 studies were reviewed and categorized into 3 groups: observational studies examining the effectiveness of operating-room cleaning and disinfections (11 studies), observational study assessing compliance with operating-room cleaning and disinfection (1 study), and interventional studies to improve operating-room cleaning and disinfection (28 studies). The SEIPS-based analysis only identified 3 observational studies examining individual work-system components influencing the effectiveness of operating-room cleaning and disinfection. Furthermore, most interventional studies addressed single work-system components, including tools and technologies (20 studies), tasks (3 studies), and organization (3 studies). Only 2 studies implemented interventions targeting multiple work-system components.

**Conclusions:**

The existing literature shows suboptimal compliance and inconsistent effectiveness of operating-room cleaning and disinfection. Improvement efforts have been largely focused on cleaning and disinfection tools and technologies and staff monitoring and training. Future research is needed (1) to systematically examine work-system factors influencing operating-room cleaning and disinfection and (2) to redesign the entire work system to optimize operating-room cleaning and disinfection.

Environmental cleaning and disinfection plays a critical role in preventing pathogen transmission and healthcare-acquired infections.^1,2^ Effective and reliable cleaning and disinfection of operating rooms is vital because of the rapid succession of patients. Pathogens from environmental reservoirs can be directly transmitted to patients or indirectly through the hands of operating-room personnel.^3^ Moreover, surgical patients are exposed to multiple invasive devices (eg, vascular and urinary catheters) and surgical wounds, facilitating microorganism invasion. A 2011 investigation identified anesthesia machine dials as vectors of bacterial contamination on vascular-access hubs.^4^ Because of the high density of hand-to-surface exposures between the environment and patients, hand hygiene alone cannot eliminate this transmission route.^5^ Adequately cleaning and disinfecting high-touch surfaces between consecutive patients is imperative.

Hospital environmental cleaning and disinfection, particularly operating-room cleaning and disinfection, is a complex process influenced by various work-system factors.^6^ Ensuring consistent and effective environmental cleaning and disinfection is challenging in everyday practice.^7–9^ We previously proposed a human-factors and systems-engineering approach based on the Systems Engineering Initiative for Patient Safety (SEIPS) model^10^ to improve hospital environmental cleaning and disinfection, particularly inpatient rooms.^6^ According to the SEIPS model, the cleaning and disinfection process is collaborative work of environmental care associates and other healthcare workers who perform different tasks (eg, cleaning high-touch surfaces, communication) with various tools and technologies (eg, cleaning tools and supplies, checklists) in a physical environment (eg, operating room size and layout) and under certain organizational conditions (eg, safety culture, work schedule). These interrelated work-system components influence the cleaning and disinfection process, which subsequently affects patients (eg, healthcare-acquired infections, patient satisfaction), healthcare workers (eg, job safety/satisfaction), and organizations (eg, reputation and reimbursement based on healthcare-acquired infection rates).^6,11^ Using this human-factors and systems-engineering approach, we conducted a systematic scoping review of empirical studies on environmental cleaning and disinfection in operating rooms to synthesize existing evidence and to identify research gaps.

## Methods

We followed the Preferred Reporting Items for Systematic Reviews and Meta-Analyses (PRISMA) guidelines.^12,13^

### Inclusion and exclusion criteria

The review was limited to peer-reviewed journal articles in English. Empirical studies on environmental cleaning and disinfection in the operating room were included. Studies were excluded if they were (1) not related to environmental cleaning and disinfection, (2) not conducted in the operating room, or (3) not empirical (eg, guidelines, review articles).

### Study search and selection

**Figure 1.**
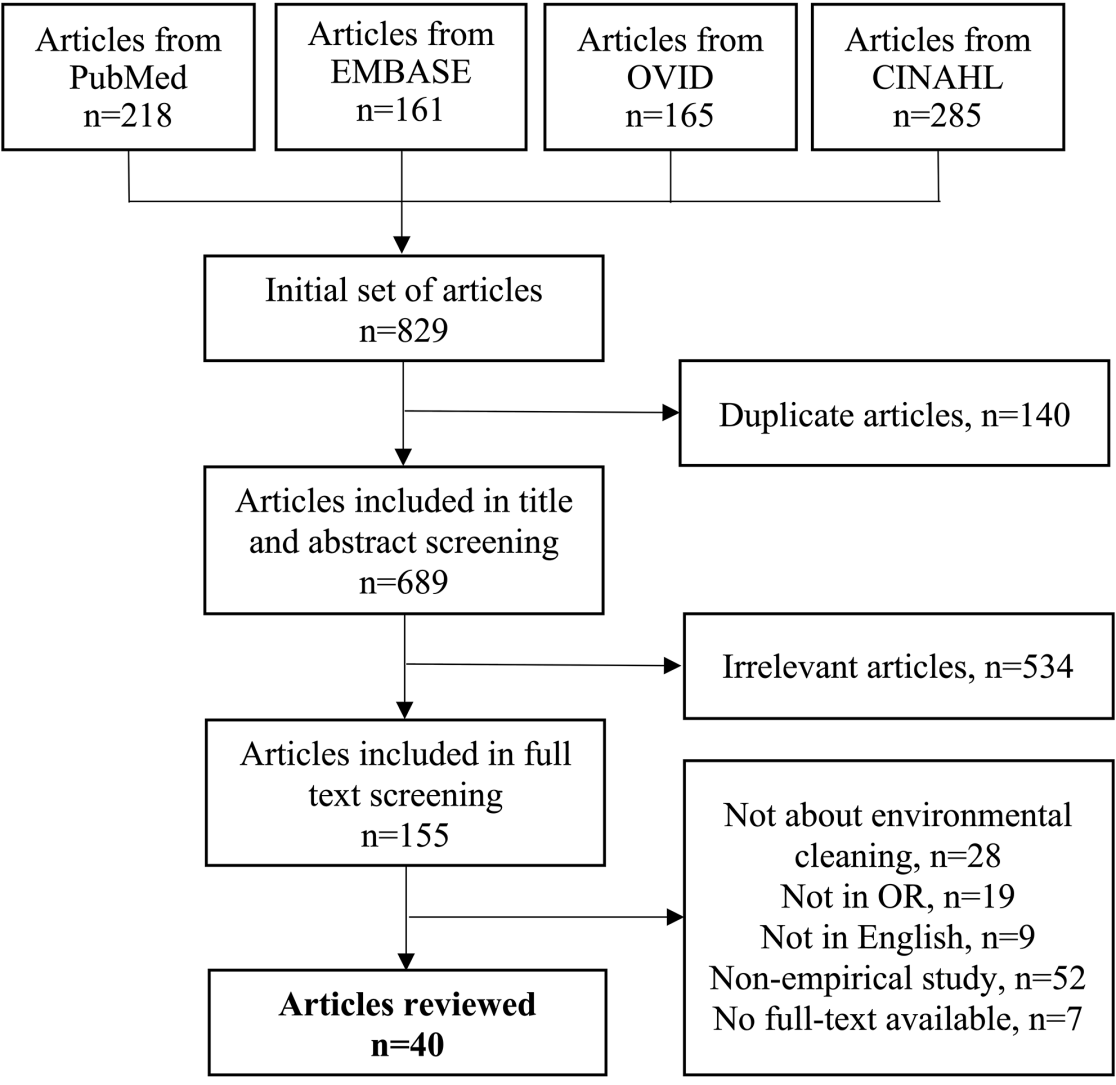
Flowchart of study search and screening.

The search was conducted in 4 databases (ie, PubMed, EMBASE, OVID, CINAHL) through February 2023. The search combined terms in 3 areas: (1) operating room (eg, operating room, operating theater, surgery), (2) environmental surface (eg, environmental, surface, floor), and (3) cleaning and disinfection (eg, cleaning, disinfection). The initial search identified 829 articles (Fig. 1). After removing 140 duplicates, 689 articles were screened for inclusion in 2 sequential steps: (1) title and abstract screening and (2) full-text screening. During each step, at least 2 researchers screened each article independently and discussed discrepancies to reach consensus. Finally, 40 articles were included for data extraction.

### Data extraction

Two researchers used a data extraction form to independently extract general information (ie, first author, title, journal, publication year) and key study characteristics (ie, country, objectives, design, outcome measures and measuring techniques, main findings, funding source). Discrepancies were discussed with a third researcher to reach consensus.

Included studies were inductively categorized into 3 groups based on their objectives and designs: (1) observational studies examining operating-room cleaning and disinfection effectiveness, (2) observational studies examining compliance with recommended operating-room cleaning and disinfection practices, and (3) interventional studies to improve operating-room cleaning and disinfection. Guided by the SEIPS model, studies were coded to identify work-system factors influencing or being modified (ie, interventions) to improve operating-room cleaning and disinfection. The included cleaning and disinfection processes (eg, turnover cleaning, terminal cleaning) and outcomes (eg, contamination, cleanliness, cleaning thoroughness) were also captured.

### Quality assessment

We used version 2 of the Cochrane risk-of-bias tool for randomized trials to assess the methodological quality of randomized controlled trials and quasi-experimental studies with nonequivalent groups design.^14^ Four researchers independently assessed each study and discussed their findings to reach consensus. Due to the lack of adequate or well-accepted quality assessment tools, the methodological quality of the remaining studies was not assessed.

## Results

Table 1 summarizes the key characteristics of the 40 studies included in this review.

**Table 1.** Characteristics of Studies Included in the Systematic Review

Characteristic	Total (N = 40)	%
**Countries**		
United States^9,15–17,22,30–32,34,35,37,38,40,41,46,47,50–55,57^	23	57.5
Italy^18,23,24,33,39^	5	12.5
United Kingdom^19,36,48^	3	7.5
Japan^45^	1	2.5
Others^20,21,25,42–44,49,56^	8	20.0
**Years**		
2020 onward^16,20,30,39,40,43,49–51,55^	10	25.0
2010–2019^9,15,17,18,21–23,25,31–36,38,41,42,44,46,47,53,56,57^	23	57.5
2000–2009^19,24,37,45,54^	5	12.5
1999 and before^48,52^	2	5.0
**Study topics and designs**		
Observational studies on operating-room cleaning and disinfection effectiveness	11	27.5
Cross-sectional design^15,16,25^	3	27.3
Case-control design^17^	1	9.1
Prospective cohort design^18–24^	7	63.6
Observational studies on operating-room cleaning and disinfection compliance	1	2.5
Cross-sectional design^9^	1	100.0
Interventional studies to improve operating-room cleaning and disinfection	28	70.0
Randomized controlled trials^40,51,52^	3	10.7
Quasi-experimental studies with nonequivalent groups design^30,31,42,43,46,49^	6	21.4
Quasi-experimental studies with pre-post design^32–39,44,45,47,50,56,57^	14	50.0
Quasi-experimental studies with interrupted time series design^48,53–55^	4	14.3
Mixed-methods design^41^	1	3.6
**Funding**		
Commercial^22,24,32–36,38,39,47^	10	25.0
Noncommercial^18–21,51^	5	12.5
Not reported^9,15–17,23,25,30,31,37,40–46,48–50,52–57^	25	62.5

### Observational studies on operating-room cleaning and disinfection effectiveness

In total, 11 observational studies examined the effectiveness of operating-room cleaning and disinfection (Table 2). Of the 11 studies, 9 assessed the impact of turnover cleaning (ie, cleaning between surgical cases) on surface contamination and cleanliness,^15–20^ air contamination,^21^ or both.^22,23^ The other 2 studies respectively assessed the impact of terminal cleaning (ie, cleaning at day’s end) on surface contamination^24^ and the impact of disinfection in the morning before the first procedure on surface and air contamination.^25^

**Table 2.** Observational Studies on the Effectiveness of Operating-Room Cleaning and Disinfection

First Author (Year)	Work System Factors^a^					Process	Outcomes	Measuring Techniques
	P	T	T&T	E	O			
Balkissoon (2014)^17^	Patient characteristics (infected vs noninfected)	…	…	…	…	Turnover cleaning	Surface contamination	Culture
Bradley (2022)^16^	…	…	…	…	…	Turnover cleaning	Surface cleanliness	ATP testing
Dallolio (2018)^23^	…	…	…	…	…	Turnover cleaning	Surface and air contamination	Culture
Dehghani (2018)^21^	…	…	…	…	…	Turnover cleaning	Air contamination	Culture
Ellis (2018)^22^	…	…	…	Surface characteristics (smooth vs irregular)	…	Turnover cleaning	Surface and air contamination, surface cleanliness	Culture, ATP testing
Frabetti (2009)^24^	…	…	…	Surface characteristics (vertical vs horizontal; smooth vs porous)	…	Terminal cleaning	Surface contamination	Culture
Griffith (2000)^19^	…	…	…	…	…	Turnover cleaning	Surface contamination and cleanliness	Culture, ATP testing, visual inspection
Matinyi (2018)^25^	…	…	…	…	…	Disinfection before first procedure	Surface and air contamination	Culture
Nascimento (2021)^20^	…	…	…	…	…	Turnover cleaning	Surface contamination and cleanliness	Culture, ATP testing, visual inspection
Richard (2017)^15^	…	…	…	…	…	Turnover cleaning	Surface cleanliness	ATP testing
Sanna (2018)^18^	…	…	…	…	…	Turnover cleaning	Surface contamination and cleanliness	Culture, ATP testing

Note. ATP, adenosine triphosphate.

a P, people; T, tasks; T&T, tools and technologies; E, environment; O, organization (based on the SEIPS model^10^).

Mixed findings were reported regarding the effectiveness of operating-room cleaning and disinfection. Although 6 studies showed that operating-room cleaning and disinfection could significantly reduce the microbiological burden of surfaces^20,21,24^ or the number of surfaces exceeding recommended minimum cleaning and disinfection levels,^17,18,23^ 5 studies showed that operating-room cleaning and disinfection did not significantly reduce the microbiological burden of surfaces^19^ or failed to reach recommended minimum cleaning and disinfection levels after cleaning.^15,16,22,25^ For example, a prospective cohort study assessing operating room turnover cleaning of 5 high-touch surfaces showed that 92% of operating rooms (via adenosine triphosphate testing) and 42% of operating rooms (via microbiological culture) had at least 1 surface that exceeded the recommended minimum cleaning and disinfection levels after cleaning.^22^

The SEIPS-based analysis identified 3 studies examining individual work-system factors that influenced the effectiveness of operating-room cleaning and disinfection. In a case–control study that examined the impact of patient infection status, standard operating-room turnover cleaning minimized surface contamination for both septic and nonseptic operations.^17^ In 2 prospective cohort studies that examined the impact of surface characteristics, the initial microbiological burden of smooth surfaces (eg, side table) and vertical surfaces (eg, wall) was low and did not significantly decrease after cleaning. However, the microbiological burden of irregular surfaces (eg, anesthesia keyboards) and horizontal surfaces (eg, floor) significantly decreased, potentially exceeding the recommended minimum cleaning and disinfection levels.^22,24^

### Observational studies on operating-room cleaning and disinfection compliance

Only 1 observational study examined compliance with recommended operating-room cleaning and disinfection practices (Table 3). Fluorescent gel markers were used to assess the thoroughness of terminal cleaning of 10 recommended high-touch surfaces in 71 operating rooms at 6 acute-care hospitals across the United States: primary and secondary over-table lights, primary and secondary operating-room doors, electrosurgery device control panel, anesthesia machine, anesthesia cart, operating room light switch, storage cabinet handle, and telephone.^9,26–29^ The overall percentage of surfaces with removed markers was low (mean, 25%; standard deviation, 15%) with wide variation across hospitals (range, 9%–50%). Work-system factors influencing operating-room cleaning and disinfection compliance were not examined.

**Table 3.** Observational Studies on Operating-room Cleaning and Disinfection Compliance

First Author (Year)	Work System Factors^a^					Process	Outcome Measures	Measuring Techniques
	P	T	T&T	E	O			
Jefferson (2011)^9^	…	…	…	…	…	Terminal cleaning	Cleaning thoroughness	Fluorescent gel marker

a P, people; T, tasks; T&T, tools and technologies; E, environment; O, organization (based on the SEIPS model^10^).

### Interventional studies to improve operating-room cleaning and disinfection

In total, 28 studies examined various interventions for improving operating-room cleaning and disinfection (Table 4). According to the SEIPS-based analysis, 20 of the 28 studies focused on tools and technologies: 12 on ultraviolet disinfection systems,^30–41^ 4 on disinfectant fogging systems,^42–45^ 2 on chemical surface disinfectants,^46,47^ and 2 on other cleaning tools.^48,49^ The remaining 8 studies investigated interventions adapting cleaning and disinfection tasks,^50–52^ implementing cleaning and disinfection audit and feedback/training systems,^53–55^ or targeting multiple work-system factors (ie, multifaceted interventions).^56,57^

**Table 4.** Interventional Studies to Improve Operating-room Cleaning and Disinfection

First Author (Year)	Work System Factors^a^					Process	Outcome Measures	Measuring Techniques
	P	T	T&T	E	O			
Armellino (2018)^53^	…	…	…	…	Remote auditing and feedback, EVC education	Terminal cleaning	Cleaning protocol adherence, cleaning duration	Remote video review
Armellino (2019)^32^	…	…	FMUV	…	…	Turnover cleaning	Surface contamination	Culture
Armellino (2020)^30^	…	…	FMUV	…	…	Terminal cleaning	Surface contamination	Culture
Bosco (2022)^39^	…	…	UVC	…	…	Turnover and terminal cleaning	Surface contamination	Culture
Casini (2019)^33^	…	…	PX-UV	…	…	Terminal cleaning	Surface contamination	Culture
ElHaddad (2017)^34^	…	…	PX-UV	…	…	Turnover cleaning	Surface contamination	Culture
Gillespie (2016)^56^	…	Cleaning frequency reduced, additional areas to clean	Microfiber and steam technology	…	Auditing and feedback, EVC and clinical staff education	Cleaning in general	Occupational health and safety, OR cost, cleaning duration, SSI	Surveillance
Green (2017)^35^	…	…	Portable PX-UV	…	…	Terminal cleaning	Surface and air contamination, HAI	Culture
Humayun (2019)^44^	…	…	HPV	…	…	Terminal cleaning	Surface contamination	Culture
Jennings (2022)^40^	…	…	UV-LED	…	…	Disinfection during surgery	Surface contamination	Culture
Lacourciere (2019)^41^	…	…	Decision support tool for determining the use of UV disinfection system	…	…	Turnover cleaning	Usefulness and acceptance of the tool	Survey
Lemmen (2015)^42^	…	…	HPV	…	…	HPV disinfection	Surface contamination	Culture
Lewis (2015)^46^	…	…	Innovative antimicrobial surface disinfectant	…	…	Terminal cleaning	Surface contamination and cleanliness	Culture, ATP testing
Loftus (2020)^51^	…	Cleaning of anesthesia machine and monitors before patient OR entry and patient admission to recovery unit	…	…	…	Cleaning in perioperative process (as part of bundle)	SSI	Surveillance
Mahida (2013)^36^	…	…	UVC	…	…	Turnover cleaning	Surface contamination	Culture
Meunier (2022)^49^	…	…	Steam cleaner	…	…	Turnover cleaning	Cleaning thoroughness	Luminol test for blood
Munoz-Price (2012)^57^	…	Cleaning of anesthesia machine by anesthesia technologists	New disinfectant	…	Auditing and feedback, EVC education	Turnover and terminal cleaning	Cleaning thoroughness, surface contamination	Fluorescent gel marker, culture
Murrell (2019)^31^	…	…	Visible-light continuous environmental disinfection system	…	…	Continuous OR disinfection	Surface contamination, SSI	Culture
Nakata (2001)^45^	…	…	AFDU with different disinfectants	…	…	Disinfection with AFDU	Surface contamination	Culture
Neil (2005)^54^	…	…	…	…	Auditing and feedback, in-situ EVC and clinical staff education	Terminal cleaning	Surface cleanliness (eg, blood, dust, sutures, paper)	Visual inspection
Parry (2022)^55^	…	…	…	…	Auditing and feedback, EVC education	Turnover and terminal cleaning	Cleaning thoroughness, HAI	Fluorescent gel marker, surveillance
Ritter (2007)^37^	…	…	Low-intensity UV with protective clothing	…	…	Disinfection during surgery	SSI	Surveillance
Simmons (2018)^38^	…	…	PX-UV	…	…	Terminal cleaning	Surface contamination	Culture
Subashini (2022)^43^	…	…	Fogging machine with different disinfectants	…	…	Disinfection with fogging machine	Surface and air contamination	Culture
Thomas (1972)^48^	…	…	Mops with detachable autoclaved heads	…	…	OR floor cleaning	Surface contamination	Culture
Wall (2022)^50^	…	Post-induction cleaning of the anesthesia workspace	…	…	…	Cleaning in perioperative process (as part of bundle)	SSI	Surveillance
Weber (1976)^52^	…	Two approaches for OR floor cleaning	…	…	…	Turnover cleaning	Surface contamination, SSI	Culture
Wiemken (2014)^47^	…	…	Improved hydrogen peroxide cleaner and disinfectant	…	…	Turnover cleaning	Cleaning thoroughness, surface cleanliness	Fluorescent gel marker, ATP

Note. ATP, adenosine triphosphate; AFDU, automatic fogging disinfection unit; EVC, environmental care; FMUV, focused multivector ultraviolet; HAI, healthcare-associated infection; HPV, hydrogen peroxide vapor; OR, operating room; PX-UV, pulsed xenon-based ultraviolet; SSI, surgical-site infection; UV, ultraviolet; UVC, ultraviolet-C; UV-LED, ultraviolet light-emitting diode.

a P, people; T, tasks; T&T, tools and technologies; E, environment; O, organization (based on the SEIPS model^10^).

#### Ultraviolet disinfection systems

Different ultraviolet disinfection systems were examined to improve operating-room disinfection, including pulsed xenon-based ultraviolet-C lights,^33–35,38,39^ continuous ultraviolet-C lights,^36^ a proprietary focused multivector ultraviolet technology,^30,32^ ultraviolet light-emitting diodes,^40^ low-intensity ultraviolet lights,^37^ and continuous near ultraviolet lights.^31^ All systems were effective in reducing surface^30–36,38–40^ and/or air^35^ contamination. Furthermore, Ritter et al^37^ reviewed 5,980 joint-replacement procedures performed by 1 surgeon over 19 years. The rate of deep infection (ie, infection deep to the fascia with a delay in wound healing or persistent discharge) was lower after replacing a horizontal laminar airflow system with a low-intensity ultraviolet light system. A mixed-methods study evaluated the acceptance and usefulness of a decision support tool for ultraviolet-C light use in turnover cleaning with patients under contact precautions.^41^

#### Disinfectant fogging systems

Two quasi-experimental studies showed that hydrogen peroxide vapor could effectively reduce the microbiological burden of different surfaces in the operating room.^42,44^ Two other quasi-experimental studies examined the use of fogging systems with different disinfectants for operating-room disinfection.^43,45^ Nakata et al^45^ assessed the effectiveness of 4 disinfectants on various bacteria: 0.5% alkyldiaminoethylglycine, 0.2% benzalkonium chloride, 0.2% sodium hypochlorite, 0.5% glutaral. The 0.2% benzalkonium chloride and 0.5% glutaral, respectively, were the most effective in reducing general bacilli and *Staphylococcus aureus* on the floor. Subashini et al^43^ compared the effectiveness of fogging 2 other disinfectants: 13% formalin, mixed solution of 0.03% polyhexamethylenebiguanide hydrochloride and 0.1% didecyl dimethyl ammonium chloride. The mixed solution was more effective than the formalin solution in reducing both surface and air contamination.

#### Chemical surface disinfectants

Two quasi-experimental studies examined the effectiveness of manual cleaning using different disinfectant products. Lewis et al^46^ examined the effectiveness of a novel antimicrobial isopropyl alcohol/organofunctional silane (IOS) solution in reducing surface contamination after terminal cleaning. Compared to non–IOS-treated sections, IOS-treated sections had a significantly lower burden of microbial contamination. Wiemken et al^47^ reported that a 1-step, ready-to-use improved hydrogen peroxide cleaner-disinfectant resulted in high cleaning thoroughness and efficacy for turnover cleaning.

#### Other cleaning tools and technologies

In response to an outbreak of gram-negative bacteria, Thomas et al^48^ introduced mops with detachable, daily autoclaved heads and a reconstructed floor-scrubbing machine with a stainless-steel tank and valve connected to the brushes to improve operating-room floor cleaning and disinfection. Other actions to reduce environmental contamination included disinfecting plumbing systems, separating clean and dirty shoe covers, improving instrument sterilizing methods, and repairing dilapidated floors, doors, and windows. Tested surfaces and wounds yielding gram-negative organisms were reduced and the outbreak was resolved. In another quasi-experimental study, Meunier et al^49^ compared the effectiveness of 3 cleaning approaches (ie, conventional, bleach followed by conventional, steam) in removing blood residues on the floor. Luminol test results showed that steam cleaning was the only approach that could completely remove blood residues.

#### Cleaning tasks

Weber et al^52^ conducted an randomized controlled trial to compare 2 approaches for turnover floor cleaning and disinfection: cleaning and disinfection between each surgical procedure (control group) and cleaning and disinfection only after contaminated or septic procedures (experimental group). Surface contamination in the control group was significantly lower than in the experimental group. Another randomized controlled trial^51^ assessed the effectiveness of a multicomponent infection prevention bundle (including frequent cleaning and disinfection of anesthesia machines and monitors), which resulted in substantial reductions in perioperative *Staphylococcus aureus* transmission (44%) and surgical site infections (88%). The infection prevention bundle was then implemented in 23 operating rooms at a large teaching hospital; 10 months after implementation, monthly bacterial transmission monitoring results were provided to anesthesia staff to optimize compliance. A quasi-experimental study with a before-and-after design showed that the introduction of the surveillance feedback significantly reduced contamination of different sites (eg, provider hand, patient skin, environmental surfaces), *Staphylococcus aureus* transmission, and surgical-site infections.^50^

#### Auditing with feedback and training

Three quasi-experimental studies examined the use of auditing with feedback and training to improve operating-room cleaning and disinfection. Neil et al^54^ used in-person auditing to assess surface cleanliness (eg, number of surfaces visibly contaminated with dust, blood, suture, or paper). Armellino et al^53^ used remote video auditing to assess cleaning protocol adherence (eg, percentage of prescribed cleaning tasks performed). Parry et al^55^ used florescent gel markers to assess cleaning thoroughness (eg, percentage of florescent gel markers removed). Feedback on auditing results was provided in different formats (eg, aggregated, individualized) to multiple stakeholders (eg, environmental care staff and manager, nurse manager, infection control specialist, organizational leaders) on various schedules (eg, daily, weekly). Environmental care staff also received a variety of training, ranging from in situ education on suboptimal operating-room cleaning and disinfection^54^ to formal training with group lessons and one-on-one coaching reviewing the features of operating-room cleaning and disinfection.^55^ Auditing with feedback and training reduced the number of visible contaminated surfaces by 97%^54^ and sustained cleaning compliance^53^ and thoroughness^55^ increased to >90%. Furthermore, improvement in cleaning thoroughness was associated with a 10-year decline in overall healthcare-acquired infection rates (by 75%), surgical-site infection rates (by 55%), and rates of hospital-acquired *C. difficile* (by 70%).^55^

#### Multifaceted interventions

Two quasi-experimental studies intervened on multiple work-system components to improve operating-room cleaning and disinfection.^56,57^ Both studies implemented auditing with feedback and training. In addition, Munoz-Price et al^57^ assigned anesthesia technologists to clean and disinfect the anesthesia machine and associated equipment between procedures and changed disinfectant from 17.2% isopropanolol to 1:10 sodium hypochloride solution; Gillespie et al^56^ reduced the frequency of floor mopping in the reception area, included additional areas on the cleaning schedule, and introduced microfiber and steam-cleaning technology. Munoz-Price et al^57^ showed that removal of florescent markers and gram-negative bacilli surface contamination improved after intervention implementation. Gillespie et al^56^ found sustained, low, deep, orthopedic surgical-site infection rates before and after intervention implementation and anecdotally discussed the impact of the intervention on occupational health and safety and cleaning.

### Quality assessment

Table 5 shows the quality assessment results of the 3 randomized controlled trials and the 6 quasi-experimental studies with nonequivalent groups design. In addition, 15 (37.5%) of the 40 studies included in this review declared a funding source: 10 had commercial funding and 5 had noncommercial funding (Table 1). Also, 7 studies receiving commercial funding investigated ultraviolet disinfection systems.^32–36,38,39^

**Table 5.** Quality Assessment Results: Risk of Bias

First Author (Year)	Random Sequence Generation	Allocation Concealment	Baseline Outcome Measurements Similar	Baseline Characteristics Similar	Incomplete Outcome Data	Knowledge of the Allocated Interventions Adequately Prevented during the Study	Protection Against Contamination	Selective Outcome Reporting	Other Risks of Bias
Armellino (2020)^30^	High	High	High	?	?	High	Low	Low	Low
Jennings (2022)^40^	Low	?	Low	Low	Low	Low	Low	Low	Low
Lemmen (2015)^42^	High	High	Low	Low	Low	High	Low	Low	Low
Lewis (2015)^46^	High	High	?	Low	Low	Low	?	Low	Low
Loftus (2020)^51^	High	?	High	High	Low	Low	High	Low	Low
Meunier (2022)^49^	High	High	High	High	High	?	Low	?	Low
Murrell (2019)^31^	High	High	Low	?	Low	High	High	Low	Low
Nakata (2001)^45^	High	High	?	High	Low	High	Low	High	Low
Weber (1976)^52^	Low	High	?	Low	Low	?	High	Low	Low

Note. ?, unclear risk of bias.

## Discussion

Effective and reliable environmental cleaning and disinfection is critical to safe operating-room operation. We conducted a systematic scoping review summarizing the current scientific literature on operating-room cleaning and disinfection. In total, 40 studies were identified, including 11 observational studies examining the effectiveness of operating-room cleaning and disinfection, 1 observational study assessing compliance with recommended operating-room cleaning and disinfection practices, and 28 interventional studies to improve operating-room cleaning and disinfection. More studies focused on turnover cleaning (n = 19) than terminal cleaning (n = 13). Studies also focused on other cleaning and disinfection processes including initial morning preprocedure disinfection, disinfection during procedures, and continuous disinfection of the operating room. Most studies were conducted in the United States (57.5%) and were published after 2010 (82.5%).

The importance of operating-room cleaning and disinfection is widely acknowledged, but evidence on the impact of operating-room cleaning and disinfection on patient outcomes is limited and inconclusive. Only 2 randomized controlled trials^51,52^ and 6 quasi-experimental studies^31,35,37,50,55,56^ examined the impact of interventions for improving operating-room cleaning and disinfection on surgical-site infections and other healthcare-acquired infections. Four studies identified a reduction in infection rates after intervention implementation,^31,37,50,51^ among which 2 implementing a multicomponent infection prevention bundle could not specify the impact of operating-room cleaning and disinfection on infection rates.^50,51^ The other 4 studies either did not find a statistically significant change in infection rates before and after intervention^35,52^ or only anecdotally discussed the impact of interventions on infection rates.^55,56^ Of 3 studies with quality assessment, 2 had high or unclear risk in 6 of 9 domains of bias.^14^ Therefore, no conclusive statement could be made regarding the impact of operating-room cleaning and disinfection on patient outcomes.

Most studies focused on the assessment of cleaning and disinfection processes and outcomes. Measures used for cleaning and disinfection process assessment included cleaning thoroughness, assessed by the removal of florescent gel markers,^9,47,49,55,57^ and cleaning protocol adherence and duration, assessed by remote video review.^53^ Assessment measures used for cleaning and disinfection outcomes included microbial contamination by microbiologic culture^17–25,30–36,38–40,42–46,48,52,57^ and cleanliness by adenosine triphosphate testing^15,16,18–20,22,46,47^ or visual inspection.^19,20,54^ Contamination may occur on surfaces that are visibly clean and, therefore, needs to be distinguished from cleanliness. Although surface contamination plays a critical role in pathogen transmission, surface cleanliness may influence occupational safety (eg, stumbling or slipping on dirty floors) and safety culture (eg, psychological effect of spreading disorder from visual soiled surfaces to disorganized work processes).^58^

Our prior work on environmental cleaning and disinfection in inpatient settings has shown that patient room cleaning and disinfection can be influenced by various work-system factors (eg, patient and family presence, interruptions).^11^ The SEIPS-based analysis only identified 3 studies examining the impact of work-system factors on operating-room cleaning and disinfection: 1 on patient characteristics (people)^17^ and 2 on surface characteristics (physical environment).^22,24^ Studies on interventions for improving operating-room cleaning and disinfection also focused on intervention effectiveness with a limited description of what and how work-system challenges to operating-room cleaning and disinfection were addressed by the interventions. Most interventions addressed single work-system components. The most frequently addressed work-system component was tools and technologies (20 studies), followed by tasks (3 studies) and organization (3 studies). Only 2 studies adapted multiple work-system components to improve operating-room cleaning and disinfection.

The most studied interventions for improving operating-room cleaning and disinfection were nontouch disinfection technologies (eg, ultraviolet disinfection systems, disinfectant fogging systems), which were expected to reduce the risk of human errors and to provide a consistent level of disinfection. Studies have demonstrated the effectiveness of nontouch disinfection technologies in reducing environmental contamination. However, they can only augment traditional manual cleaning because of their limitations in removing (in)organic matters (eg, blood, dust).^59^ Also, there remains a lack of understanding of the practical challenges to the use of these nontouch disinfection technologies for operating-room disinfection (eg, interruption of clinical workflow, prolonged cycle time, inadequate staff training). Hence, their application in practice has been limited.^60^

Of 40 studies, 31 included in this review were observational studies or quasi-experimental studies with pre-post or interrupted time-series designs. The scientific rigor of the 3 randomized controlled trials^40,51,52^ and 6 quasi-experimental studies with nonequivalent groups design^30,31,42,43,46,49^ was low to moderate. In addition, 25 studies included in this review did not reveal their funding sources and 10 were commercially funded, which might have introduced a desirability bias.

In conclusion, this systematic scoping review summarizes the current literature on operating-room cleaning and disinfection. The included studies with diverse scopes, aims, and methods provided inconsistent evidence on the effectiveness of operating-room cleaning and disinfection. To demonstrate the importance of operating-room cleaning and disinfection, more studies are needed to examine its impact on patient (eg, prevention of surgical site infections and other healthcare-acquired infections), employee (eg, job satisfaction, fatigue, and burnout of environmental care associates), and organizational (eg, reputation and reimbursement based on healthcare-acquired infection rates) outcomes. These studies provided evidence on suboptimal compliance with recommended operating-room cleaning and disinfection practices. Future research needs to systematically examine work-system facilitators and barriers to operating-room cleaning and disinfection. Moreover, effective and sustainable interventions for improving operating-room cleaning and disinfection (eg, novel cleaning and disinfection technologies, environmental care monitoring and training programs) should consider the broader work systems. Furthermore, increased noncommercial funding is needed to support future research on operating-room cleaning and disinfection.
